# Measuring patient disability in the peri‐operative period using the WHODAS 2.0 questionnaire: a systematic review

**DOI:** 10.1111/anae.70287

**Published:** 2026-07-24

**Authors:** Romijn M. Boerlage, Daniël van Dartel, Jacqueline E. M. Vernooij, Nick J. Koning, Linda M. Posthuma, Cor J. Kalkman, Carine J. M. Doggen, Benedikt Preckel

**Affiliations:** ^1^ Department of Anaesthesiology and Pain Medicine Rijnstate Hospital Arnhem the Netherlands; ^2^ Department of Health Technology and Services Research Technical Medical Centre, University of Twente Enschede the Netherlands; ^3^ Clinical Research Center, Rijnstate Hospital Arnhem the Netherlands; ^4^ Department of Anaesthesiology Amsterdam UMC location AMC Amsterdam the Netherlands; ^5^ Department of Intensive Care Gelre ziekenhuizen Apeldoorn the Netherlands; ^6^ Department of Intensive Care Albert Schweitzer Hospital Dordrecht the Netherlands; ^7^ Department of Anaesthesiology University Medical Centre Utrecht Utrecht the Netherlands; ^8^ Amsterdam Public Health, Quality of Care Research Institute Amsterdam the Netherlands; ^9^ Amsterdam Cardiovascular Science Research Institute Amsterdam the Netherlands

**Keywords:** disability, patient‐reported outcome measure, peri‐operative medicine, WHODAS 2.0, World Health Organization

## Abstract

**Introduction:**

The World Health Organization Disability Assessment Schedule 2.0 (WHODAS 2.0) is recommended by peri‐operative core outcome sets as a measure of disability. Despite its growing use, the effect of different surgical procedures on disability measured by WHODAS 2.0 remains poorly understood and heterogeneous reporting across studies limits direct comparison.

**Methods:**

We conducted a systematic review to assess the effect of different surgical procedures on disability measured by WHODAS 2.0, and the reporting of disability endpoints in peri‐operative medicine. The primary outcome was the difference in WHODAS 2.0 summary score before and after surgery. Disability scores were calculated as a metric between 0% (no disability) and 100% (complete disability).

**Results:**

Thirty‐nine studies were included. Pre‐operative disability ranged from 2% to 61%. In 21 studies, disability increased by 1% to 17% within 3 months of surgery, with little change between 6 and 12 months (0% to ‐2%). Studies of orthopaedic surgery generally reported high pre‐operative disability (> 35%) but also reductions in disability (10–37% reduction) within 3 months of surgery. In contrast, studies evaluating cancer surgery (n = 12) generally reported lower pre‐operative disability (< 20%) but small increases (1–8%) within 3 months of surgery. Cardiovascular surgical studies displayed pre‐operative disability between 4% and 17% with a difference in disability of ‐8% to +3% 3 months after surgery. Three different thresholds to define disability and 13 different WHODAS 2.0 endpoints were used, measured at eight different time‐points after surgery.

**Discussion:**

The effect of surgical procedures on postoperative disability as measured by WHODAS 2.0 is influenced by the type of surgery. Orthopaedic surgery may decrease disability, whereas cancer surgery may be associated with persistent increases. Standardised reporting of disability endpoints is needed to enhance the quality and comparability of future studies using WHODAS 2.0.

## Plain Language Summary

**What we did:**

Researchers looked at studies that used a questionnaire called the World Health Organization Disability Assessment Schedule 2.0 (WHODAS 2.0). This questionnaire measures how much difficulty people have with everyday activities. The researchers reviewed studies of different types of surgery and compared patients' disability levels before and after their operations.

**Why did we do it:**

Doctors often use WHODAS 2.0 to see how surgery affects patients' lives, but results from different studies can be difficult to compare. The researchers wanted to understand how disability changes after surgery and whether different types of surgery have different effects on recovery.

**What we found:**

The researchers found that the effect of surgery on disability depends on the type of operation. People having orthopaedic surgery, such as operations on bones and joints, usually had less disability after surgery and often showed the biggest improvements. People having cancer surgery often had a small increase in disability after surgery. For heart and blood vessel surgery, disability levels were usually similar before and after the operation. The researchers also found that studies measured disability in different ways, making it hard to compare results. They concluded that using the same methods in future studies would help doctors better understand how surgery affects patients' lives.

## Introduction

Patient‐reported outcome measures capture health status, quality of life and functioning directly from the perspective of patients and complement traditional clinical endpoints. The World Health Organization Disability Assessment Schedule 2.0 (WHODAS 2.0) questionnaire was developed as a general patient‐reported outcome measure to measure disability and global functioning across different diseases and populations [1]. Disability, as measured by WHODAS 2.0, is recommended as a standardised patient‐centred endpoint in peri‐operative clinical trials [2]. WHODAS 2.0 has good psychometric properties, is well validated and is applied broadly in clinical research [1, 3]. Over the past 15 years, the use of WHODAS 2.0 has increased in surgical trials and peri‐operative clinical practice [3]. WHODAS 2.0 has been validated in surgical populations with a minimal clinically important difference of 5% [4, 5].

Disability is present frequently before surgery and may follow different trajectories, contributing to heterogeneity in surgical populations [4, 5]. Measuring disability before and after surgery allows changes in functional status to be assessed over time [6]. Accounting for pre‐operative individual differences using longitudinal analysis can reduce unexplained variability in postoperative outcomes and improve statistical efficiency [7, 8]. Recent guidelines recommend completing WHODAS 2.0 before surgery to inform patients of the risks of disability after surgery, although high‐quality evidence supporting this approach is lacking [9].

Despite the increasing use of WHODAS 2.0, the effect of different types of surgical interventions on disability over time remains unclear. In addition, reporting of how WHODAS 2.0 is used and reported varies between studies, making direct comparison of studies difficult. Therefore, the aims of this systematic review were to analyse the effect of different surgical procedures on patient disability measured using the WHODAS 2.0 and assess how disability endpoints are reported in the peri‐operative literature.

## Methods

This systematic review was reported according to the PRISMA statement [10].

WHODAS 2.0 assesses functionality across six domains: cognition; mobility; self‐care; getting along; life activities; and participation [1]. It is available as a full version with 36 questions (36‐item); short version with 12 questions (12‐item); and hybrid of the full and short version (12 + 24‐items). Items assess difficulties experienced during the previous 30 days, with responses ranging from ‘none’ to ‘cannot do’ [6]. Metric summary scores range from 0% (no disability) to 100% (full disability) and are calculated using the complex scoring method [6]. Detailed information on WHODAS 2.0 is in online Supporting Information Appendix S1. WHODAS 2.0 measures the degree of disability and therefore no strict cut‐off points for significant disability were defined in the initial publications [1, 6, 11, 12].

We searched MEDLINE, PubMed Central, Embase, Scopus and the Cochrane Library for relevant articles. Two healthcare librarians contributed to the development and adaptation of the search strategy, and a third librarian independently peer reviewed the search strategy using the Peer Review of Electronic Search Strategies checklist [13]. Citations of key articles were checked to confirm that the search strategy was appropriate to not miss relevant articles [3, 4, 5, 14]. The final search was run from inception of the databases until 22 December 2025. Full search strategies for each database are in online Supporting Information Appendix S2.

Eligible studies were randomised controlled trials and observational studies that reported WHODAS 2.0 scores both before and after surgery in adults aged ≥ 18 y. Reviews, meta‐analyses, case reports, case series, conference papers and conference proceedings were excluded, as were studies without an abstract in English or Dutch languages. Two independent reviewers performed screening and selection through Rayyan (Rayyan Systems Inc., Cambridge, MA, USA) [15]. Disagreements were resolved through discussion and consensus; a third reviewer adjudicated unresolved cases. The primary outcome was the difference in WHODAS 2.0 summary scores before and after surgical intervention. Other secondary outcomes included: reported WHODAS 2.0 endpoints; censoring due to missingness or death; use of WHODAS 2.0 endpoints as a primary or secondary endpoint; and the methods for applying and reporting WHODAS 2.0 in surgical trials.

Data were extracted by one study member and checked by a second study member using a pre‐piloted data extraction form (online Supporting Information Appendix S3) in Covidence (Veritas Health Innovation, Melbourne, VIC, Australia). Data were extracted for both the analysed total study population and any predefined study subgroups (e.g. cohorts, treatment arms of clusters). Risk of bias for the primary outcome was assessed independently by two researchers using the Cochrane Collaboration Risk Of Bias In Non‐Randomised studies of interventions (ROBINS‐I) tool, as the study aimed to assess the effect of surgery on patient disability [16].

Results are presented for the total study population unless otherwise specified. For studies reporting different subsets, results are presented per subset. Frequencies and percentages were weighted and aggregated across subsets to estimate results for the total study population. WHODAS 2.0 scores are presented as mean or median scores, depending on the availability of data. The scores were visualised using graphs and harvest plots to display disability over time. These plots are descriptive and intended to summarise the available evidence. The standard deviation, if not reported, was estimated if sufficient other data were available (e.g. 95%CI) using established methods [17]. Metric scores were calculated accordingly for studies reporting sum scores. Studies with uninterpretable WHODAS 2.0 scores (e.g. unclear scale or calculation) were excluded. Changes in disability were calculated as the average difference between pre‐operative and postoperative WHODAS 2.0 metric scores within each subset. The prevalence of disability per time‐point was extracted when reported (frequency, percentage and total sample size). Missing values were derived from other available data within the study. For studies reporting only the incidence of patients without disability (i.e. WHODAS 2.0 score < 16% or < 25%), values were inverted to estimate the prevalence of disability. All analyses were performed in R version 4.3.3 (RStudio Team, Boston, MA, USA).

## Results

Our search identified 1568 unique articles, of which 90 articles were eligible. Thirty‐five articles were pooled into 12 studies as they included the same study populations, resulting in 67 eligible studies. Thirty‐nine studies were included in the analysis (Fig. 1) [18, 19, 20, 21, 22, 23, 24, 25, 26, 27, 28, 29, 30, 31, 32, 33, 34, 35, 36, 37, 38, 39, 40, 41, 42, 43, 44, 45, 46, 47, 48, 49, 50, 51, 52, 53, 54, 55, 56, 57, 58, 59, 60].

**Figure 1:**
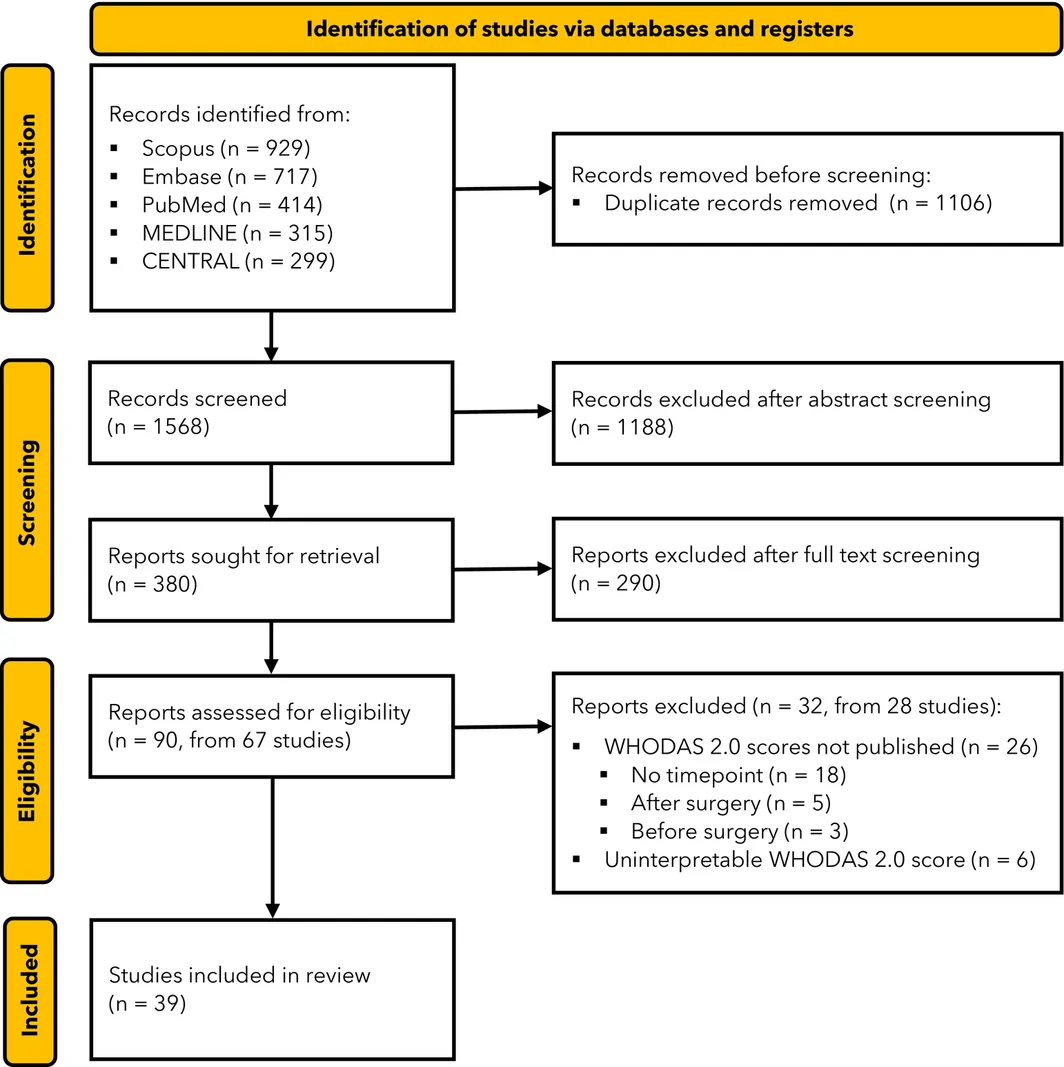
Study flow diagram.

Characteristics of the 39 included studies are shown in Table 1; 15 were randomised controlled trials and 24 were observational. Twenty‐three studies included studies from a single surgical subspecialty, most commonly cardiac (five studies) and orthopaedic (four studies). Of the remaining studies with mixed surgical populations, only one reported WHODAS 2.0 endpoints by type of surgery. Almost one‐third of studies (12 studies) focused on cancer surgery. The sample sizes ranged from 24 [19, 29] to 6644 patients [49]. Additional characteristics per study population are in online Supporting Information Tables S1–S3. All studies included both sexes except for one study of radical prostatectomies [54]. In 35 studies the average age of the study population was ≥ 50 y, with 22 studies reporting a mean age ≥ 65 y. The prevalence of pre‐operative disability, defined as WHODAS 2.0 score ≥ 25%, ranged from 12% to 90% [25, 26, 32].

**Table 1:** Summary characteristics of all included studies.

	Included studies
	n = 39
**Study design**	
Randomised controlled trial	15
Prospective cohort study	23
Retrospective cohort study	1
**Study population**	
Cancer surgery	12
Cardiovascular surgery	6
‐ Cardiac	5
‐ Vascular	1
Orthopaedic surgery	4
Neurosurgery	3
Bariatric surgery	1
Mixed surgery	13
**Sample size**	
< 100	11
100–499	17
500–999	6
≥ 1000	5
**Postoperative measurements included in primary analysis; months**	
1	13
2	2
3	24
6	8
12	6
60	1

Risk of bias assessments are shown in online Supporting Information Figures S1 and S2. Missing postoperative WHODAS 2.0 data due to nonresponse was the main contributor to serious risk of bias in 20 studies, with missing data otherwise ranging from 0% to 71%. Only two studies reported complete data [29, 45], whilst two provided no information on completeness [20, 48]. Missing data were most commonly handled using available‐case analysis (23 studies) followed by complete case analysis (10 studies). The remaining studies used: multiple imputation (five studies); linear mixed modelling (one study); worst‐case analysis (one study); or a combination of all three (one study). Mortality was reported in 21 studies. Seven studies performed a worst‐case analysis where death was assigned a WHODAS 2.0 metric score of 100%; three did not study patients who died from the analysis; and two used available‐case analysis. Handling of mortality data was not described explicitly in nine studies.

Of the included 39 studies, 18 reported WHODAS 2.0 data for the total study population and 21 for study subsets (44 subsets). Average scores are in Fig. 2 and online Supporting Information Table S4. Pre‐operative WHODAS 2.0 scores ranged from 2% in a mixed elective major abdominal surgery population [44] to 61% in patients at high risk of developing chronic postsurgical pain [18]. Average WHODAS 2.0 scores within 2 months of surgery ranged from 6% to 30% (14 studies). At 3 months, WHODAS 2.0 scores ranged from 1% to 53% (24 studies); at 6 months from 6% to 50% (eight studies); and at 12 months from 4% to 20% (six studies). Two studies reporting both 6 and 12‐month WHODAS 2.0 scores showed no or minimal change (0% to ‐2%) between these time‐points.

**Figure 2:**
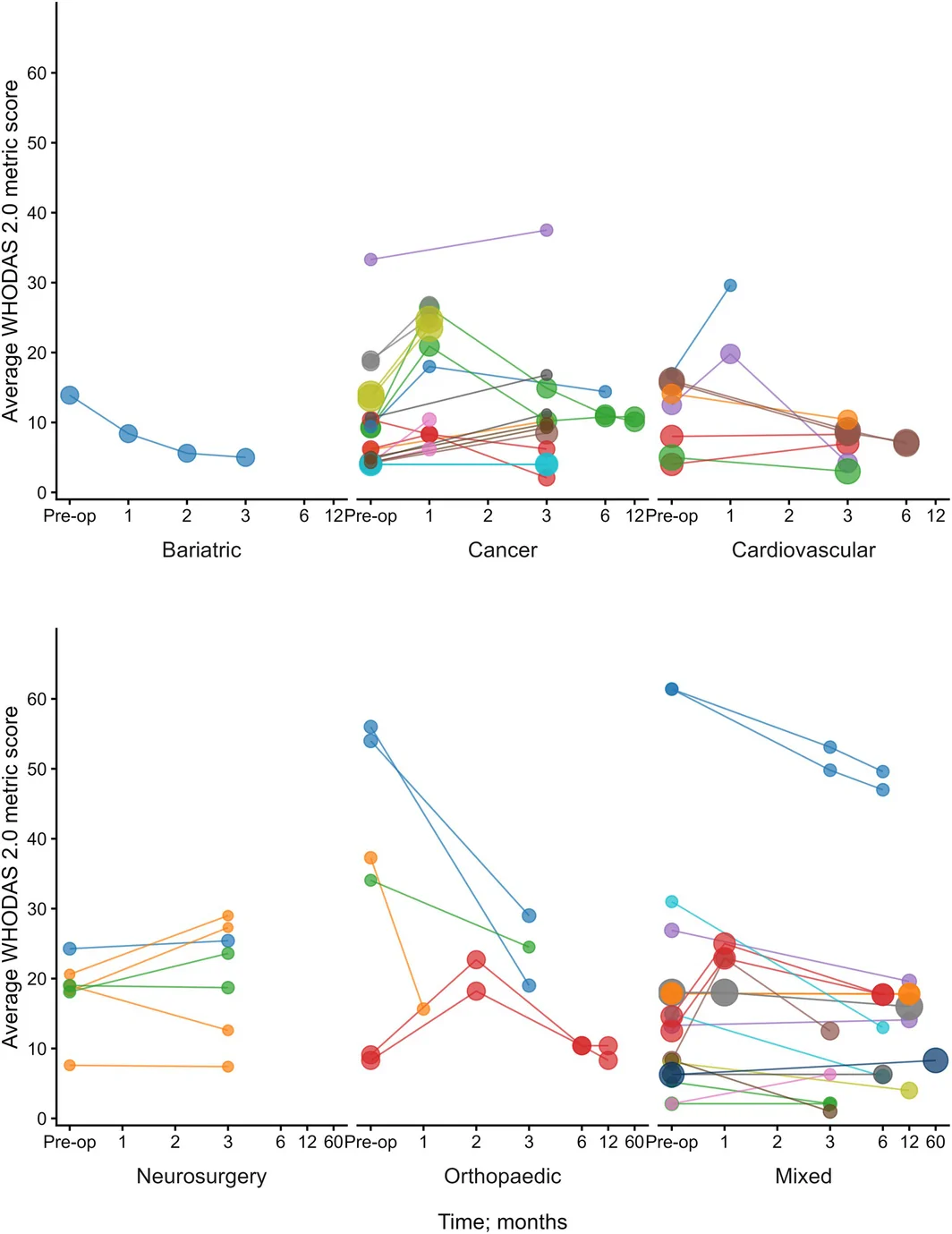
Average WHODAS 2.0 metric scores (0–100%) per type of surgery. Every dot represents the total study population (18 studies) or study subset, i.e. cohort, group or cluster (21 studies), depending on data availability. Per surgical type, each colour represents one study. The total number of patients (sample size) is shown as increasing size of the dot: < 50; 50–99; 100–499; 500–999; and ≥ 1000. Time in months represents the number of months after surgery. Pre‐op; pre‐operatively.

Change in average WHODAS 2.0 score over time is displayed in Fig. 3 and online Supporting Information Figures S3 and S4. Compared with pre‐operative average scores, disability increased within 2 months in 11 studies (+1% to +17%); was unchanged in one; and decreased in three (‐1% to ‐22%). At 3 months, scores were similar in seven studies; higher in 12 studies (+1% to +9%); and lower in three studies (‐1% to ‐37%). Change in average scores ranged from ‐18% to +5% at 6 months (eight studies) and ‐7% to +2% at 12 months (six studies). One study with a 60‐month follow‐up reported a small increase of 2% when compared with pre‐operative disability [59].

**Figure 3:**
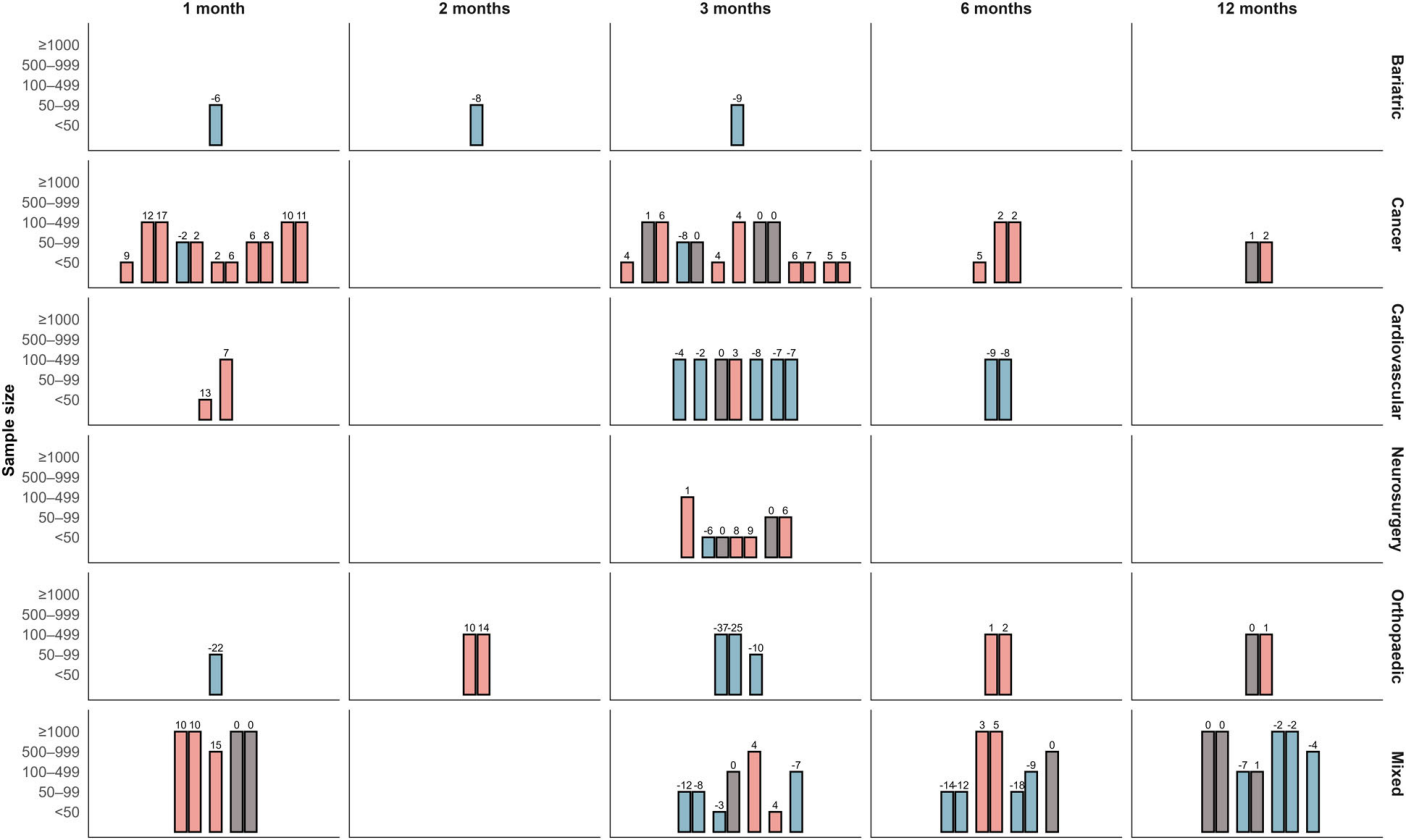
Change in average WHODAS 2.0 metric score (0–100%) compared with before surgery, grouped by type of surgical intervention (rows) and timing of measurement after surgery (columns) until 12 months. Each bar represents a single measurement for either the total population (18 studies) or study subset, i.e. cohort, group or cluster (21 studies), depending on data availability. Study subsets from one single study are nested together. Bar height reflects the sample‐size categorised (five levels). The direction of change in average WHODAS 2.0 score difference is grouped as: blue, decrease ≤ ‐1.0%; red, increase ≥ 1.0%; grey, no change > ‐1.0% and < 1.0. Each bar is labelled with the corresponding change in average WHODAS 2.0 score compared to before surgery, rounded to the nearest whole number.

In patients undergoing orthopaedic surgery, three studies (458 patients) reported lower average WHODAS 2.0 scores within 3 months of surgery, with a maximum decrease in score of ‐37% in the orthopaedic trauma population [25]. In contrast, 10/12 studies in patients undergoing cancer surgery (1896 patients) reported higher disability scores at all postoperative time‐points when compared with pre‐operative scores, with a maximum average increase of +17% at 1 month. In one study on patients undergoing laparoscopic colectomy, small reductions in WHODAS scores were observed (‐1% to ‐4%) compared with pre‐operative scores [22], while one study in patients undergoing colorectal cancer surgery reported no changes [40]. Similarly, the majority of neurosurgical studies showed an increase in average WHODAS 2.0 score after surgery. Cardiovascular surgical studies showed contrasting results 3 months after surgery, with an average WHODAS 2.0 score difference of ‐8% vs. +3%. A sensitivity analysis stratified by study design (randomised vs. observational) showed similar patterns across surgical types (online Supporting Information Figure S5), with the exception of one randomised trial in patients undergoing hip fracture surgery which reported higher postoperative WHODAS 2.0 scores 2 months after surgery [25].

Disability endpoints and WHODAS 2.0 methodology are shown in Table 2. In 22 studies, WHODAS 2.0 formed part of the primary outcome; 32 studies used the 12‐item version; three used the 36‐item version; and four did not specify which version was used. WHODAS 2.0 was administered at eight different postoperative time‐points, of which 1 month (17 studies); 3 months (28 studies); and 6 months (10 studies) were most common. Twenty‐one studies measured disability only once after surgery; 21 studies did not define disability thresholds; 13 defined relevant disability as WHODAS 2.0 score ≥ 25%; and four defined ‘clinically significant disability’ ≥ 35% [5]. In total, 13 different WHODAS 2.0 endpoints were reported.

**Table 2:** Disability thresholds and method of measuring WHODAS 2.0 in included studies.

	Included studies
	n = 39
**Definition: disability as a threshold**	
Disability defined	17
Metric score > 25%	13
Clinically significant disability: metric score > 35%	4
**Definition: no disability as a threshold**	
Metric score < 16%	2
**Definition: minimal clinically important difference**	
5% difference in metric score	8
8% difference in metric score	6
**Study endpoint**	
(Included in) primary study endpoint	22
**Study endpoint**	
WHODAS 2.0 score	39
Disability	8
Disability‐free‐survival*	10
New disability (or death)	10
Change in disability	5
**Administration method**	
Self‐reported	8
Interview	9
Interview and self‐reported	5
Interview of patient or by‐proxy	2
Not specified	15
**Measurements after surgery** **	
1	21
2	11
3 or 4	7
**Postoperative measurements; months** **	
1	17
2	2
3	28
6	10
12	6
Other	3

* Composite endpoint of mortality and disability.

** Not all measurements were available for inclusion in the primary analysis.

## Discussion

Our key findings include: WHODAS 2.0 disability scores, when compared with pre‐operative scores, often increased within the first 2 months of surgery and remained largely stable between 6 and 12 months; disability trajectories differed according to type of surgery, with patients undergoing orthopaedic surgery reporting high pre‐operative disability and marked improvement after surgery, whereas patients undergoing cancer surgery more often showed postoperative increases with limited return to pre‐operative levels; and pre‐operative disability (WHODAS 2.0 ≥ 25%) was common with prevalence ranging from 12% to 90%. Reporting was variable, with three different thresholds to define disability and 13 different endpoints used across studies. Missing WHODAS 2.0 data contributed to increased risk of bias in over half of included studies, with > 15% of data missing at one time‐point in 14 studies. Statistical handling of death also varied.

An important finding of our review is the considerable variation in patient‐reported disability over time between different surgical populations. In orthopaedic surgery, functional limitation is often the motivation for surgery and represents the primary target for improvement. Accordingly, studies frequently reported high pre‐operative disability and substantial improvement within 1–3 months of surgery [25, 27, 45, 55]. These findings should be interpreted cautiously due to high rates of missing postoperative data in most studies, although similar improvements in function and pain have been reported in other orthopaedic surgery studies using patient‐reported outcome measures [61, 62].

Major cancer surgery is often performed with curative or life‐prolonging intention but may result in functional loss. WHODAS 2.0 may therefore capture temporary disability related to surgery and neoadjuvant treatments, as well as permanent functional limitations from life‐preserving procedures (e.g. oesophagectomy, permanent stoma, limb amputation). These factors likely contributed to the observed increase in disability within 3 months of surgery and the lack of recovery to pre‐operative levels in included studies [20, 21, 29, 31, 33, 37, 53, 54]. Heterogeneity in cancer types and treatment pathways are also likely to have contributed to wide variation in pre‐operative disability and limits direct comparison of postoperative trajectories. Nevertheless, in homogeneous cancer populations, WHODAS 2.0 may inform shared decision‐making by balancing survival benefit against functional loss after surgery. Cardiac surgery aims to improve survival and prevent adverse outcomes but may result in new postoperative disability due to complications [5]. As such, WHODAS 2.0 may capture functional limitations to surgical recovery and/or complications. We found that disability increased at 1 month and subsequently improved by 3 months, but these findings should be interpreted cautiously due to serious to critical risk of bias in three out of five cardiac studies [32, 34, 41].

Pooling WHODAS 2.0 outcomes across heterogeneous populations may obscure meaningful disability patterns. A study including over 6600 surgical patients reported no change in average disability at 30 days after surgery and minimal decline of average disability after 1 year [49]. However, such averages may mask distinctive disability time trends per type of surgery as shown in this review. Future studies using WHODAS 2.0 should compare populations with similar surgical aims or stratify analyses by surgical type. We found that WHODAS 2.0 was measured at seven different time‐points after surgery, which precluded meta‐analysis. Pre‐operative WHODAS 2.0 measurement remains essential because baseline disability varies widely and patients are their own comparator [6].

For postoperative WHODAS 2.0 measurements, it is important to choose the timing based on study aim and provide sufficient support, as these measurements demand a significant amount of resource and time. WHODAS 2.0 measurement 3 months after surgery may provide the optimal time‐point, especially in studies focusing on the final result of surgery. Evidence suggests that functional recovery of major surgery typically takes 3–6 months [63]. Also, measuring at 3 months may capture more procedure‐related events than earlier measurements [64, 65, 66]. In line with these suggestions, across the majority of included studies, WHODAS 2.0 was measured 3 months after surgery. However, measuring disability again 6 months after surgery may offer insights into longer‐term recovery. Between 6 and 12 months after surgery, WHODAS 2.0 scores yield similar results (Figs. 2 and 3) [63]. Unnecessary late measurements of WHODAS 2.0 may lengthen a study and risk a higher percentage of missing data due to nonresponse. Further research is needed to clarify whether 6 months is an adequate alternative to 12 months after surgery. For now, standardised measurements of WHODAS 2.0 at a minimum of 3 months and optional 6 months after surgery may provide optimal insights into functional recovery while preserving comparability of research.

Currently, the various definitions of disability and variety of WHODAS 2.0 endpoints used, limit the interpretability and comparability of published studies. As a result of WHODAS 2.0 scores not being published along the other disability endpoints, only 59% of eligible studies could be included in the current analysis. In the 34 included studies, thresholds of 8%, 25%, 5%, 16% and 35% were used, of which only the last three thresholds are validated in a surgical population [4]. Increased use of thresholds and composite endpoints cumulates the number of WHODAS 2.0 endpoints quickly. This situation bears some similarity to the issue of ‘intra‐operative hypotension’ as a possible contributor to adverse postoperative outcomes in peri‐operative studies. When different research groups each continue using their own – different – definitions and thresholds, this hampers the interpretation of observed associations between peri‐operative events and disability [67]. A recent study on the prevalence of peri‐operative disability as measured by WHODAS 2.0 found small numbers of studies using the same disability thresholds and time‐points [68]. Clearly, consensus on definitions and measurement time‐points can improve the quality and generalisability of peri‐operative outcome studies. The WHODAS 2.0 metric score is a cross‐cultural validated outcome with accurate display of disability at that time. A combination of the metric score with an absolute value displaying change in disability over time per patient (e.g. minimal clinically important difference, new disability), can meaningfully represent disability for both research and clinical practice.

The use of WHODAS 2.0 in peri‐operative research frequently leads to missing data due to patient nonresponse and death, yet details on how these missing values were addressed are often lacking. Although response rates have been described as acceptable, most studies show high percentages of missing data which are far above acceptable rates [69, 70]. Missing data is a recurrent challenge in patient‐reported outcome measure research and may, for WHODAS 2.0, lead to a misrepresentation of overall measured disability. For instance, disability may be underestimated when more disabled patients fail to respond or deceased patients are excluded from the analyses. A study using complete case analysis may have lowered the average disability of a population by removing deceased patients, who had the most severe outcomes [48]. Conversely, disability may be overestimated when fully recovered patients do not respond or when death is scored 100%. A study using a worst‐case analysis may have increased the average disability of the population by assuming the maximum disability is equivalent to death [41].

To help interpret measured disability, it is essential to provide a clear description of the number, characteristics and cause of patients with missing data at each time‐point. Furthermore, characteristics (e.g. pre‐operative WHODAS 2.0 scores) of patient groups with missing data should be compared to those without. Multiple imputation methods, apart from simple imputation, are used rarely but may in some cases provide a solution [6, 71]. To address missing data due to death, recent literature recommends joint‐longitudinal survival models or available‐case analysis, as these methods are less susceptible to distort measured disability [23]. However, these models are only appropriate when disability has been assessed at multiple time‐points after surgery, highlighting the value of measuring WHODAS 2.0 repeatedly in the postoperative period. With only one postoperative measurement available, the optimal method for analysing death remains undefined. Altogether, potential overestimation or underestimation of disability due to nonresponse or death should be carefully considered when choosing disability as a study endpoint and when interpreting disability outcomes.

WHODAS 2.0 was published in combination with a manual to improve standardisation of use. Given the considerable variation in how WHODAS 2.0 was used and reported across studies, we propose a set of practical recommendations to improve standardisation and reporting in future peri‐operative research (Table 3). Understanding peri‐operative disability outcomes may contribute meaningfully to personalised patient counselling; tailored rehabilitation planning; healthcare resource allocation; and risk stratification. While certain disability trends may be predicted based on the type of surgery, additional factors also have a significant impact. Associations have been identified between disability and multiple factors, such as frailty; age; blood loss volume; nutritional status; and postsurgical pain [35, 60, 63, 70]. Two studies on surgical lobectomy for lung cancer with similar population profiles besides location‐bound differences showed the same increase in disability 3 months after surgery (+4%), but pre‐operative disability varied considerably (WHODAS score average 33% vs. 6%) [20, 29]. Furthermore, evidence on disability over time among patients who do not undergo surgery remains limited and therefore restricts conclusions to patients who had surgery. Nevertheless, individually expected disability trends may be valuable information to discuss with the patient when deciding on various treatment options [72]. It remains to be investigated whether and how current established disability trends reported in clinical studies can be translated to the individual patient.

**Table 3:** Proposed recommendations for application and display of WHODAS 2.0 in surgical trials.

**Timing of measurements**
‐ At least before surgery and 3 months after surgery
**WHODAS 2.0 endpoints**
‐ Publish a threshold or composite WHODAS 2.0 endpoint always in combination with WHODAS 2.0 metric scores to enhance comparability ‐ In mixed surgical populations, display WHODAS 2.0 results by type of surgery/aim of surgery, in addition to disability results for the entire study population
**Calculate**
‐ Calculate the WHODAS 2.0 12‐item version as metric summary score ranging from 0 (no disability) to 100 (full disability).
**Missing data**
‐ Report number of patients with missing measurements per time‐point ‐ In case of high rates of missing data due to patients not responding, provide and compare patient characteristics for patients with and without WHODAS 2.0 as supplemental and consider imputation methods ‐ In case of death, report the method used to handle death ‐ Preferable methods of handling death when disability is measured repeatedly postoperative: available‐case analysis (analysing all available data) or joint‐longitudinal models
**Describe**
Provide information in the main publication (or supplemental file) on at least the following topics:
‐ Version used (12‐item, 36‐item or 12 + 24‐item) ‐ Administration method: keep method of administration per patient the same as much as possible ‐ Timing of measurements ‐ Calculation method and value of WHODAS 2.0 score (sum score or metric summary score) ‐ Cite at least one publication as required by the World Health Organization [1, 6, 11]

Our review has strength and limitations. Only studies with WHODAS 2.0 measurements before and after surgery, as recommended by the WHODAS 2.0 manual and guidelines, and studies available in English or Dutch language were included, restricting conclusions to studies with both these measurements. However, results should be interpreted with caution due to high percentage of missing WHODAS 2.0 data in the included studies. Clinical diversity (e.g. differences in timing of measurements after surgery, patient populations within each surgical category and peri‐operative care) and methodological diversity (e.g. differences in administration method and handling of missing data) were considered too high for a meaningful meta‐analysis to be performed. Our study provides a comprehensive first overview of WHODAS 2.0 scores across peri‐operative studies, highlighting the influence of surgical type on disability trajectories. In addition, critical evaluation of disability endpoints and their limitations led to practical recommendations to improve the use and interpretation of disability as measured by WHODAS 2.0. These proposed recommendations may represent an initial step toward a formal consensus or Delphi process.

In conclusion, the time‐trend of postoperative disability as measured sequentially by WHODAS 2.0 is influenced by the type of surgery. Orthopaedic surgery was often associated with reduced patient‐reported disability after surgery, whereas cancer surgery frequently showed a persistent increase in disability. To improve the quality and comparability of peri‐operative studies using WHODAS 2.0, further work is needed to standardise reporting of endpoints, such as measurements before and 3 months after surgery, metric scores and threshold or composite disability endpoints.

## Supporting information


**Appendix S1.** WHODAS 2.0.
**Appendix S2.** Search strategies.
**Appendix S3.** Data extraction form.

**Table S1.** Detailed characteristics of included studies.
**Table S2.** Population characteristics of included studies.
**Table S3.** Surgical procedure types in studies with mixed surgical populations.
**Table S4.** Pre‐operative and postoperative average WHODAS 2.0 metric scores per study.

**Figure S1.** Risk of bias assessment summary.
**Figure S2.** Risk of bias assessment per study.
**Figure S3.** Postoperative average WHODAS 2.0 scores and change from pre‐operative baseline.
**Figure S4.** Risk of bias and change in average WHODAS 2.0 score from pre‐operative baseline.
**Figure S5.** Sensitivity analysis stratified by study design.
