# Supplementary material for: Measuring patient disability in the peri‐operative period using the WHODAS 2.0 questionnaire: a systematic review
Source: Anaesthesia. 2026 Jul 24;81(11):1569–80. doi: 10.1111/anae.70287 (PMC13659789; doi:10.1111/anae.70287)
Supplement: Supplementary file 1 [file ANAE-81-1569-s001.docx]

**Appendix S1: WHODAS 2.0**

**Content**
WHODAS 2.0 is a generic instrument to measure health and disability levels in individuals.[1] WHODAS 2.0 was developed in accordance with the International Classification of Functioning Disability and Health (ICF), the international standard to describe and measure health and disability. ICF defines disability as “an umbrella term for impairments, activity limitations and participation restrictions”.[2] Disability is not distinguished by different health conditions, but is a complex interaction between health conditions and contextual factors (i.e. personal and environmental factors).[2,3] To measure the degree of disability, WHODAS 2.0 differentiates six domains for which functionality is scored: cognition, mobility, self-care, getting along, life activities and participation. WHODAS 2.0 was developed and validated in cross-cultural studies resulting in a disability measure sensitive to change, independent of patients’ cultural background. Through the years, WHODAS 2.0 has been translated into 47 languages and used in more than 90 countries.[4]

WHODAS 2.0 is available as a full version with 36 questions (36-item), short version with 12 questions (12-item) and hybrid of the full version and short version (12+24-items). Every item states examples of difficulties with functioning during the previous 30 days, with response options ranging from “none”, “mild”, “moderate”, “severe”, and “extreme to “cannot do”. The 36-item version is most detailed and can produce a summary score as well as a score per domain. The 12-item version produces only a summary score, explains 81% of the variance of the 36-item version and is quicker to administer than the 36-item version. The 12+24-item version uses the 12-item version to screen for domains with problematic functioning. Depending on the answers of the 12 items, additional items, with a maximum of 24, will be asked. The 36-item version and the 12-item version can be administered by the individuals themselves, by an interviewer or by a relative (proxy-administered). The dynamic 12+24 version can only be administered by an interviewer or by computer-adaptive testing.[3]

**Scoring**
The WHODAS 2.0 manual gives two options to calculate summary scores for the 12-item and 36-item WHODAS 2.0 versions, simple scoring and complex scoring. Simple scoring scores each item 1 to 5 and sums all items. This results in a maximum score of 60 in the 12-item version, and 180 for the 36-item version. Simple scoring is a useful method in clinical practice but less appropriate to compare across populations.[3] Complex scoring, or the ‘item-response-theory’, is suitable for more precise analyses across populations and subpopulations. In the 36-item version, complex scoring assigns a score of 0 to 4 or 0 to 2 per item and calculates a metric score. To calculate the metric summary score, all items are summed up and divided by the maximum possible score of 106, resulting in a metric ranging from 0 (no disability) to 100 (full disability). Domain metric scores can be calculated in the 36-item version by dividing the sum score per domain by the maximum possible score per domain.[3]

For the 12-item version, the WHODAS 2.0 manual does not specify a method for complex scoring. An alternative simple scoring method, calculating metric summary score ranging from 0 (no disability) to 100 (full disability), was described by Andrews and colleagues.[5] Since the introduction of WHODAS 2.0, the alternative method has regularly been used.[6-9] This method sums the score of all 12 items, each ranging from 0 to 4, and divides the total by the maximum possible score of 48. The 12-item metric summary score ranges from 0 to 100 and can be compared to metric summary scores of the 36-item version when comparing populations or patients.

Missing responses of items can be substituted through various imputation methods.[3] Maximum one (12-item) or two (36-item) missing responses can be substituted by taking the mean of the other items (simple imputation). In case of more missing values and with enough background variables available, “hot deck” imputation or multiple imputation can be used. Hot deck imputation fills-in missing responses by using observed values from matched, randomly selected participants with complete data from the same data set.[10,11] Through multiple imputation, missing responses are replaced by multiple plausible values, analysed separately per completed dataset and then pooled to reflect uncertainty.[12]

**Defining disability**
WHODAS 2.0 measures the degree of disability and therefore no strict cut-off points for significant disability were defined in the initial publications.[1-3,5] Andrews et al., using the 12-item version with summary scores between 0 and 48, stated that patients in the top 10% who all scored between 10 and 48, were likely to have clinically significant disability.[5] Shulman et al., using the 12-item version in surgical patients, classified the binary endpoints disability by a metric score of at least 25% and new disability as an increase of 8% compared to the preoperative score.[13] In a later publication, these authors described a minimal clinically important difference of 5% in surgical patients and they defined a score less than 16% to be ‘disability-free’, and a score of at least 35% to be ‘at least moderate clinically significant disability’.[8]

**References**

1.	Ustün TB, Chatterji S, Kostanjsek N, et al. Developing the world health organization disability assessment schedule 2.0. *Bull World Health Organ* 2010; **88:** 815-23. <https://doi.org/10.2471/blt.09.067231>

2.	. International classification of functioning, disability and health : Icf*.* Geneva: World Health Organization, 2001.

3.	Ustun TB, Kostanjesek N, Chatterji S, Rehm J. *Measuring health and disability: Manual for who disability assessment schedule whodas 2.0*, 1st edn.: World Health Organization, 2010.

4.	Federici S, Bracalenti M, Meloni F, Luciano JV. World health organization disability assessment schedule 2.0: An international systematic review. *Disabil Rehabil* 2017; **39:** 2347-80. <https://doi.org/10.1080/09638288.2016.1223177>

5.	Andrews G, Kemp A, Sunderland M, Von Korff M, Ustun TB. Normative data for the 12 item who disability assessment schedule 2.0. *PLoS One* 2009; **4:** e8343. <https://doi.org/10.1371/journal.pone.0008343>

6.	Shulman MA, Myles PS, Chan MTV, McIlroy DR, Wallace S, Ponsford J. Measurement of disability-free survival after surgery. *Anesthesiology* 2015; **122:** 524-36. <https://doi.org/10.1097/ALN.0000000000000586>

7.	McIsaac DI, Taljaard M, Bryson GL, et al. Frailty and long-term postoperative disability trajectories: A prospective multicentre cohort study. *British Journal of Anaesthesia* 2020; **125:** 704-11. <https://doi.org/10.1016/j.bja.2020.07.003>

8.	Shulman MA, Kasza J, Myles PS. Defining the minimal clinically important difference and patient-acceptable symptom state score for disability assessment in surgical patients. *Anesthesiology* 2020; **132:** 1362-70. <https://dx.doi.org/10.1097/ALN.0000000000003240>

9.	Myles P, Bellomo R, Corcoran T, et al. Restrictive versus liberal fluid therapy in major abdominal surgery (relief): Rationale and design for a multicentre randomised trial. *BMJ Open* 2017; **7:** e015358. <https://dx.doi.org/10.1136/bmjopen-2016-015358>

10.	Ford B. *An overview of hot-deck procedures.*, 78 edn. New York: Academic Press, 1983.

11.	Andridge RR, Little RJA. A review of hot deck imputation for survey non-response. *International Statistical Review* 2010; **78:** 40-64. <https://doi.org/10.1111/j.1751-5823.2010.00103.x>

12.	Rubin DB. *Multiple imputation for nonresponse in surveys*: Wiley, 1987.

13.	Shulman M, Myles P. Measuring perioperative outcome. *Curr Opin Anaesthesiol* 2016; **29:** 733-8. <https://doi.org/10.1097/aco.0000000000000383>

**Appendix S2: Search strategy**

**Research question**

What is the effect of surgical interventions on the difference between preoperative and postoperative disability in surgical patients as measured by WHODAS 2.0?

**Additional information search strategy and study selection**

To ensure no articles were missed due to abstracts not mentioning WHODAS or WHODAS results, the search strategy was extended by searching reference lists. Users of WHODAS 2.0 are required to properly cite the World Health Organization (WHO) as the source [1]. Therefore, an extended search in SCOPUS was based on required and commonly used references when citing WHODAS 2.0 [2-7]. The search results were downloaded and stored using the Endnote X9 software [8]. Duplicate records were manually removed by matching for title, authors, and year of publication.

A two-phase screening process was used to determine the eligibility of studies found. In the first phase, titles and abstracts were screened for eligibility. All articles not excluded in the first phase, and articles found through reference checking in Scopus, were screened for full text in the second phase.

## **Summary of search results**

| Database: | 22-12-2025 |
| --- | --- |
| MEDLINE, (PubMed) | 414 |
| MEDLINE (via Ovid) | 315 |
| EMBASE (via Ovid) | 717 |
| Cochrane library  *reviews*  *Trials*  *protocols* | 299  101  185  13 |
| SCOPUS | 929 |
| Total before deduplication | 2674 |
| Total after deduplication | 1568 |

## **Search terms**

| **Term 1** | | | | |
| --- | --- | --- | --- | --- |
| **WHO Disability Assessment Schedule** | | | | **WHODAS endpoints** |
| WHODAS / WHO DAS | | | | New disabilit* |
|  | | | | Disability free survival |
| WHO | Disability | | | Clinically significant disability |
| World health organisation |  | | | Patient reported disability |
| World Health Organization |  | | | New onset disability |
|  | | | |  |
| WHO | Disability | | Schedule |  |
| World Health Organisation |  | | Assessment |  |
| World Health Organization |  | | Scale |  |
|  |  | | Measurement |  |
|  |  | |  |  |
| **Reference in SCOPUS** | | | | |
| [2-4], other validation studies e.g. [9,10] | | *REF ("whodas" OR "who-das" OR "who-disability" OR "world-health-organi?ation-disability") OR REF ("world-health-organi?ation" AND ("disability-assessment" OR "disability-schedule" OR "disability-scale") )* | | |
| [5] | | *REF ((Shulman) AND (“minimal clinically important difference”) AND (disabilit*))* | | |
| [6] | | *REF ((Shulman) AND (“predicting death or disability”))* | | |
| [7] | | *REF ((Shulman) AND (“measurement of disability-free survival”))* | | |

**Term 2**

**Surgery**

Anaesthe*	(Anaesthetic, anaesthesiology, anaesthesia, ...)

Anesthe*

Surger* (surgeries, surgical, surgery)

Surgeon*

Surgical*

Operation

Operative

Perioperative

Peri-operative

preoperative

Pre-operative

intraoperative

Intra-operative

postoperative

Post-operative

Incisi*

Extracti*

Excisi*

Ostectom*

Osteotom*

Coronoid*

invasive*

restorati*

**References**

1.	World Health O. Copyright, licensing and permissions, 2025. <https://www.who.int/about/policies/publishing/copyright> (accessed December 17, 2025/.

2.	Andrews G, Kemp A, Sunderland M, Von Korff M, Ustun TB. Normative data for the 12 item who disability assessment schedule 2.0. *PLoS One* 2009; **4:** e8343. <https://doi.org/10.1371/journal.pone.0008343>

3.	Ustun TB, Kostanjesek N, Chatterji S, Rehm J. *Measuring health and disability: Manual for who disability assessment schedule whodas 2.0*, 1st edn.: World Health Organization, 2010.

4.	Ustün TB, Chatterji S, Kostanjsek N, et al. Developing the world health organization disability assessment schedule 2.0. *Bull World Health Organ* 2010; **88:** 815-23. <https://doi.org/10.2471/blt.09.067231>

5.	Shulman MA, Myles PS, Chan MTV, McIlroy DR, Wallace S, Ponsford J. Measurement of disability-free survival after surgery. *Anesthesiology* 2015; **122:** 524-36. <https://doi.org/10.1097/ALN.0000000000000586>

6.	Shulman MA, Kasza J, Myles PS. Defining the minimal clinically important difference and patient-acceptable symptom state score for disability assessment in surgical patients. *Anesthesiology* 2020; **132:** 1362-70. <https://dx.doi.org/10.1097/ALN.0000000000003240>

7.	Shulman MA, Wallace S, Gilbert A, Reilly JR, Kasza J, Myles PS. Predicting death or disability after surgery in the older adult. *Anesthesiology* 2023; **139:** 420-31. <https://doi.org/10.1097/aln.0000000000004683>

8.	The EndNote Team. Endnote*.* Philadelphia, PA: Clarivate, 2013.

9.	Schiavolin S, Ferroli P, Acerbi F, et al. Disability in italian neurosurgical patients: Validity of the 12-item world health organization disability assessment schedule. *International journal of rehabilitation research. Internationale Zeitschrift fur Rehabilitationsforschung. Revue internationale de recherches de readaptation* 2014; **37:** 267-70. <https://dx.doi.org/10.1097/MRR.0000000000000064>

10.	Ida M, Naito Y, Tanaka Y, Matsunari Y, Inoue S, Kawaguchi M. Feasibility, reliability, and validity of the japanese version of the 12-item world health organization disability assessment schedule-2 in preoperative patients. *Journal of Anesthesia* 2017; **31:** 539-44. <https://dx.doi.org/10.1007/s00540-017-2349-8>

## **Search queries**

### Medline/Pubmed

| # | Searches – Abstract | 22-12-2025 |
| --- | --- | --- |
| 1 | "whodas"[All Fields] OR "who-das"[All Fields] OR "whodisability"[All Fields] OR "who-disability"[All Fields] OR "world-health-organisation-disability"[All Fields] OR "world-health-organization-disability"[All Fields] | 1720 |
| 2 | "world health organization"[MeSH Terms] | 41,717 |
| 3 | "world-health-organization"[All Fields] OR "world-health-organisation"[All Fields] | 157,423 |
| 4 | "disability-assessment"[All Fields] OR "disability-schedule"[All Fields] OR “disability-scale”[All Fields] | 5,697 |
| 5 | (#2 OR #3) AND #4 | 1,134 |
| 6 | "new disabilit*"[All Fields] OR "disability-free survival"[All Fields] OR "clinical* significant disability"[All Fields] OR "patient reported disability"[All Fields] OR "new onset disability"[All Fields] | 573 |
| 7 | #1 OR #5 OR #6 | 2,341 |
| 8 | anesthesiology[MeSH Terms] OR anesthesia[MeSH Terms] OR "perioperative medicine"[MeSH Terms] OR "specialties, surgical"[MeSH Terms] OR "surgical procedures, operative"[MeSH Terms] OR "surgery, oral"[MeSH Terms] OR osteotomy[MeSH Terms] OR "orthognathic surgical procedures"[MeSH Terms] OR "preoperative period"[MeSH Terms] OR "intraoperative period"[MeSH Terms] OR “perioperative period” [MeSH Terms] "postoperative period" [MeSH Terms] OR "preoperative care"[MeSH Terms] OR "perioperative care"[MeSH Terms] OR "intraoperative care”[MeSH Terms] OR “postoperative care”[MeSH Terms] | 225,037 |
| 9 | "anesthe*"[All Fields] OR "anaesthe*"[All Fields] OR "surger*"[All Fields] OR "surgical*"[All Fields] OR "surgeon*"[All Fields] OR "operation*"[All Fields] OR “operative*”[All Fields] OR "perioperati*"[All Fields] OR “incisi*”[All Fields] OR “extracti*”[All Fields] OR “excisi*”[All Fields] OR “ostectom*”[All Fields] OR osteotom*[All Fields] OR coronoid*[All Fields] OR invasive*[All Fields] OR restorati*[All Fields] OR “preoperati*”[All Fields] OR “intraoperati*”[All Fields] OR “postoperati*”[All Fields] | 7,371,417 |
| 10 | #8 OR #9 | 7,371,958 |
| 11 | #7 AND #10 | 414 |

### Medline (Ovid)

| # | Searches - Abstract | 22-12-2025 |
| --- | --- | --- |
| 1 | (whodas OR who das OR who disability OR world health organi#ation disability).mp | 1,719 |
| 2 | exp world health organization/ | 41,735 |
| 3 | world health organi#ation.mp | 124,939 |
| 4 | (disability assessment OR disability schedule OR disability scale).mp | 5,707 |
| 5 | 2 OR 3 | 125,590 |
| 6 | 4 AND 5 | 1,081 |
| 7 | (disability free survival OR new disabilit$ OR clinical$ significant disabilit$ OR patient reported disabilit$ OR new onset disabilit$).mp | 586 |
| 8 | 1 OR 6 OR 7 | 2,333 |
| 9 | exp anesthesiology/ or exp anesthesia/ or exp perioperative medicine/ or exp specialties, surgical/ or exp surgical procedures, operative/ or exp surgery, oral/ or exp osteotomy/ or exp orthognathic surgical procedures/ or exp perioperative period/ or exp preoperative period/ or exp intraoperative period/ or exp postoperative period/ or exp preoperative care/ or exp perioperative care/ or exp intraoperative care/ or exp postoperative care/ | 4,076,796 |
| 10 | (an?esthe$ OR surger$ OR surgeries OR surgical$ OR surgeon$ OR operation$ OR operative$ OR perioperati$ OR incisi$ OR extracti$ OR excisi$ OR ostectom$ OR osteotom$ OR coronoid$ OR invasive$ or restorati$ OR preoperati$ OR intraoperati$ OR postoperati$).mp | 5,983,566 |
| 11 | 9 OR 10 | 7,349,994 |
| 12 | 8 AND 11 | 315 |

### Embase (ovid)

| # | Searches | 22-12-2025 |
| --- | --- | --- |
| 1 | (whodas OR who das OR who disability OR world health organi#ation disability).mp |  |
| 2 | exp world health organization/ |  |
| 3 | world health organi#ation.mp |  |
| 4 | (disability assessment OR disability schedule OR disability scale).mp |  |
| 5 | 2 OR 3 |  |
| 6 | 4 AND 5 | 2,305 |
| 7 | (disability free survival OR new disabilit$ OR clinical$ significant disabilit$ OR patient reported disabilit$ OR new onset disabilit$).mp | 985 |
| 8 | 1 OR 6 OR 7 | 3,857 |
| 9 | exp anesthesiology/ or exp anesthesia/ or exp perioperative medicine/ or exp specialties, surgical/ or exp surgical procedures, operative/ or exp surgery, oral/ or exp osteotomy/ or exp orthognathic surgical procedures/ or exp perioperative period/ or exp preoperative period/ or exp intraoperative period/ or exp postoperative period/ or exp preoperative care/ or exp perioperative care/ or exp intraoperative care/ or exp postoperative care/ | 7,063,604 |
| 10 | (an?esthe$ OR surger$ OR surgeries OR surgical$ OR surgeon$ OR operation$ OR operative$ OR perioperati$ OR incisi$ OR extracti$ OR excisi$ OR ostectom$ OR osteotom$ OR coronoid$ OR invasive$ or restorati$ OR preoperati$ OR intraoperati$ OR postoperati$).mp | 8,380,991 |
| 11 | 9 OR 10 | 10,282,300 |
| 12 | 8 AND 11 | 717 |

### Cochrane

| # | Searches | 22-12-2025 |
| --- | --- | --- |
| 1 | whodas OR who NEXT das OR "who disability" OR (world NEXT health NEXT organi?ation NEXT disability) | 802 |
| 2 | [mh "world health organization"] | 493 |
| 3 | world NEXT health NEXT organi?ation | 17698 |
| 4 | "disability assessment" OR "disability assessment schedule" OR “disability assessment scale” | 1838 |
| 5 | (#2 OR #3) AND #4 | 482 |
| 6 | (disabilit* NEXT free NEXT survival) OR (new NEXT disabilit*) OR (new NEXT onset NEXT disabilit*) OR (clinical* NEXT significant NEXT disabiit*) OR (patient NEXT reported NEXT disabilit*) | 169 |
| 7 | #1 OR #5 OR #6 | 1037 |
| 8 | [mh anesthesiology] OR [mh anesthesia] OR [mh “perioperative medicine”] OR [mh “specialties, surgical”] OR [mh “surgical procedures, operative”] OR [mh “surgery, oral”] OR [mh osteotomy] OR [mh “orthognathic surgical procedures”] OR [mh “preoperative period”] OR [mh “intraoperative period”] OR [mh “postoperative period”] OR [mh “perioperative period”] OR [mh “preoperative care”] OR [mh “perioperative care”] OR [mh “intraoperative care”] OR [mh “postoperative care”] | 187,230 |
| 9 | an?esthe* OR surger* OR surgeries OR surgical* OR surgeon* OR operation* OR operative* OR perioperati* OR incisi* OR extracti* OR excisi* OR ostectom* OR osteotom* OR coronoid* OR invasive* or restorati* OR preoperati* OR intraoperati* OR postoperati* | 550,071 |
| 10 | #8 or #9 | 590,633 |
| 11 | #7 and #10 | 299 |
|  | Cochrane reviews:  Cochrane Protocols:  Trials: | 101  13  185 |

### Scopus

| # | Search - Abstract | 22-12-2025 |
| --- | --- | --- |
| 1 | TITLE-ABS-KEY("whodas" OR "who-das" OR "who-disability" OR "world-health-organi?ation-disability") | 2,273 |
| 2 | TITLE-ABS-KEY("world-health-organi?ation") | 223,036 |
| 3 | TITLE-ABS-KEY ("disability-assessment" OR "disability-schedule" OR "disability-scale") | 11,603 |
| 4 | *#2 AND #3* | 1,829 |
| 5 | TITLE-ABS-KEY ("new-disabilit*" OR "disabilit* free survival" OR “new onset disabilit*” OR “clinical* significant disabilit*” OR “patient reported disabilit*”) | 806 |
| 6 | *REF ("whodas" OR "who-das" OR "who-disability" OR "world-health-organi?ation-disability")* | 7,112 |
| 7 | *REF ("world-health-organi?ation" AND ("disability-assessment" OR "disability-schedule" OR "disability-scale") )* | 3,834 |
| 8 | *REF ((Shulman) AND (“minimal clinically important difference”) AND (disabilit*))* | 93 |
| 9 | *REF ((Shulman) AND (“predicting death or disability”) AND (disabilit*))* | 20 |
| 10 | *REF ((Shulman) AND (“measurement of disability-free survival”))* | 204 |
| 11 | *#1 OR #4 OR #5 OR #6 OR #7 OR #8 OR #9 OR #10* | 8,546 |
| 12 | TITLE-ABS-KEY("anesthe*" OR "anaesthe*" OR "surger*" OR "surgical*" OR "surgeon*" OR "operation*" OR "operative*" OR "perioperati*" OR "incisi*" OR "extracti*" OR "excisi*" OR "ostectom*" OR "osteotom*" OR "coronoid*" OR "invasive*" OR "restorati*" OR "preoperati*" OR "intraoperati*" OR "postoperati*" OR "pre-operati*" OR "intra-operati*" OR "post-operati*" OR "peri-operati*") | 11,694,247 |
| 13 | *#11 AND #12* | 929 |

**Appendix S3: Data extraction form**

Data extraction was performed using the online tool Covidence systematic review software® (Veritas Health Innovation, Melbourne, Australia. Available at www.covidence.org)
