# Supplementary material for: Measuring patient disability in the peri‐operative period using the WHODAS 2.0 questionnaire: a systematic review
Source: Anaesthesia. 2026 Jul 24;81(11):1569–80. doi: 10.1111/anae.70287 (PMC13659789; doi:10.1111/anae.70287)
Supplement: Supplementary file 2 [file ANAE-81-1569-s002.docx]

**Figure S1:** Risk of bias assessment per domain in 39 included studies according to the ROBINS-I.

**Figure S2:** Risk of bias assessment per study according to the ROBINS-I, categorized as low risk (+), moderate risk (-), serious risk (x) and critical risk (!). Domains: D1: bias due to confounding. D2: Bias due to selection of participants; D3: Bias in classification of interventions; D4 Bias due to deviations from intended interventions; D5: Bias due to missing data; D6 Bias in measurement of outcomes; D7 Bias in selection of reported result.

**Figure S3:** Change in average WHODAS 2.0 metric scores from preoperative baseline to postoperative follow-up (y-axis) plotted against the corresponding average WHODAS 2.0 metric scores (x-axis) at 1, 2, 3, 6, and 12 months after surgery. Data are presented as medians with interquartile ranges (square symbols) or means with standard deviations (circular symbols). Error bars are omitted when measures of variability (IQR or SD) were unavailable. Each symbol represents a single measurement for either the total population (18 studies) or study subset, i.e. cohort, arm or cluster (21 studies), depending on the availability of data. Surgical type is indicated by colour: cardiovascular (blue), orthopaedic (orange), cancer (green), neurosurgery (purple), bariatric (grey), and mixed (brown).

**Figure S4:** Harvest plot displaying change in average WHODAS 2.0 metric score (0% to 100%) compared to before surgery, grouped by type of surgical intervention (rows) and timing of measurement after surgery (columns) until 12 months. Each bar represents a single measurement for either the total population (18 studies) or study subset, i.e. cohort, arm or cluster (21 studies), depending on the availability of data. Study subsets from one single study are nested together. Bar height reflects the overall risk of bias assessed using the Cochrane Collaboration’s Risk Of Bias In Non-Randomised studies of interventions (ROBINS-I) tool [16]. The direction of change in average WHODAS 2.0 score is grouped as decrease (blue; score difference ≤-1.0%), increase (red; score difference ≥1.0%) or no change (grey; score difference >-1.0% and <1.0) in disability, and is visualised by colour of the bar. Each bar is labelled with the corresponding change in average WHODAS 2.0 score compared to before surgery, rounded to the nearest whole number.

**Figure S5:** Average WHODAS 2.0 metric scores (0% to 100%) per type of surgery, stratified by study design: observational (above) and randomised (below). Every dot represents the total study population (study-level) or study subset, i.e. cohort, arm or cluster (subset-level), depending on the availability of data. Per surgical type, each colour represents one study. The total number of patients (sample size) included in the dot is shown as size of the dot categorised in 5 levels (<50, 50-99, 100-499, 500-999, ≥1000). Preop stands for preoperatively; Time in months represents the number of months after surgery.
