# Supplementary material for: Measuring patient disability in the peri‐operative period using the WHODAS 2.0 questionnaire: a systematic review
Source: Anaesthesia. 2026 Jul 24;81(11):1569–80. doi: 10.1111/anae.70287 (PMC13659789; doi:10.1111/anae.70287)
Supplement: Supplementary file 3 [file ANAE-81-1569-s003.docx]

Table S1: Study characteristics of 39 included studies

| **Author (year)** | **Design** | **Surgical intervention** | **Total population or cohorts** | **Total sample size** | **version** | **Administration method** | **Handling of death** | **% non-response** § |
| --- | --- | --- | --- | --- | --- | --- | --- | --- |
| Admiraal (2023) [19] | Prospective cohort | Elective surgery | Standard care (n=84)/transitional pain service (n=92) | 176 | 12-item | Not specified | Available data analysis | 0%-6% |
| Amirpour (2025) [20] ¶¶ | Prospective cohort | abdominal surgery (83% cancer surgery) | Total population | 24 | 12-item | Not specified | Not described, no deaths | 0%-29% |
| Bobcikova (2023) [21] | Prospective cohort | lung resection | Total population | 44 | 36-item | Interview | Not described, no deaths | Unclear |
| Catto (2022) [22] | RCT | radical cystectomy | Open (n=156) /robotic  (n=161) | 317 | 12-item | Self-reported | Not described, death occurred | 15%-45% |
| Collange (2024) [23] | RCT | laparoscopic colectomy | Opioid-free (n=78)/Opioid-sparing (n=77) anaesthesia | 155 | 12-item | Interview | Not described, no deaths | 3% |
| Daza (2024)* [24,25] | RCT | major abdominal surgery | Liberal (n=1493)/ restrictive (n=1490) fluid protocol | 3000 | 12-item | Interview of patient or proxy | Available data analysis | 5% |
| Edgley (2019) [26] | Prospective cohort | orthopaedic trauma (poly- or monotrauma) surgery | With (n=171) /without (n=132) severe acute pain at PACU | 303 | 12-item | Interview and self-reported | Not described, no deaths | 24% |
| Ferraz (2018) [27] | Prospective cohort | elective non-cardiac, non-obstetric, and non-neurological surgery; | With (n=24) /without PQR (n=158) | 182 | 12-item | Not specified | Not described, no deaths | 0%-23% |
| Galli (2018) [28] | Prospective cohort | elective hip prostheses and elective knee prostheses | Total population | 80 | 36-item** | Interview and self-reported | Censored as missing | 0%-1% |
| Haakseth (2023) [29] | Prospective cohort | endovascular aneurysm repair | Total population | 25 | 36-item† | Interview and self-reported | Not described, no deaths | 12%-24% |
| Ida (2019) [30] | Prospective cohort | video-assisted thoracoscopic surgical lobectomy | Total population | 24 | 12-item | Self-reported | Not described, no deaths | 0% |
| Kant (2025) [31] | RCT | Elective noncardiac surgery | Proactive blood pressure management (n=1618)/ usual management (n=1629) | 3247 | 12-item | Self-reported | Scored 100% | 6%-30% |
| Kinugasa (2023) [32] | Prospective cohort | elective major abdominal surgery | Total population | 260 | 12-item | Not specified | Scored 100% | 0%-12% |
| Lee (2025) [33] §§ | RCT | cardiac surgery | Total population | 143 | 12-item | Not specified | Scored 100% | 12%-18% |
| Liu (2020) [34] | RCT | video-assisted thoracoscopic surgical lobectomy (cancer) | Prehabilitation program (n=37)/standard care (n=36) | 73 | 12-item | Interview | Not described, no deaths | 6% |
| Ma (2025) [35] | Prospective cohort | elective open-heart surgery (cardiac) | Total population | 351 | 12-item | Interview | Available data analysis | 0%-7% |
| McIsaac (2020)* [7,36] | Prospective cohort | elective, major inpatient noncardiac surgery | Frailty  (n=252)/no Frailty (n=435) | 702 | 12-item | Interview | Scored 100% | 0%-8% |
| McIsaac (2022) [37] | RCT | elective surgery for intra-abdominal and intrathoracic cancer | Prehabilitation (n=94)/ standard care (n=88) | 204 | 12-item | Interview | Not described, no deaths | 10% |
| McIsaac (2025) [38] | RCT | elective, inpatient head and neck, thoracic, abdominal, pelvic, or vascular surgery (75% cancer surgery) ‡‡ | Prehabilitation (n=423)/ usual care (n=424) | 847 | 12-item | Not specified | Scored 100% | 0%-8% |
| Miles (2022) [39] | Prospective cohort | elective cardiac surgery | Iron deplete  (n=239)/iron replete (n=239) | 480 | 12-item‡ | Not specified | Not described, death occurred | 0.4%-5% |
| Miles (2025) [40] | Prospective cohort | colorectal cancer surgery (minimal invasive vs major) | Iron deficient (n=170)/iron replete (n=250) before surgery | 420 | Not specified | Interview | Not described, no deaths | 0%-1% |
| Milne (2022) [41] | Prospective cohort | elective cardiac surgery | Total population | 146 | 12-item | Not specified | Scored 100% | 0%-14% |
| Parke (2021) [42] | RCT | cardiac surgery | Usual care (n=357)/fluid administration protocol (n=358) | 715 | 12-item | Interview and self-reported | Not described, death occurred | 1%-11% |
| Posthuma (2022) [43] | RCT | noncardiac, intermediate or high-risk surgery | Total population | 747 | 12-item | Self-reported | Available data analysis | 1%-21% |
| Sato (2025) [44] | Prospective cohort | urological abdominal surgery, lower gastrointestinal surgery, thoracic surgery, oral and maxillofacial surgery | Total population | 46 | Not specified | Not specified | Not described, no deaths | 2% |
| Schiavolin (2015) [45] | Prospective cohort | cervical or lumbar laminectomy and discectomy | Total population | 75 | 12-item | Not specified | Not described, no deaths | 0%-0% †† |
| Schiavolin (2018) [46] | Prospective cohort | neurosurgical procedure for brain tumor | Total population | 101 | 12-item | Not specified | Excluded from analysis | 8%-30% |
| Schiavolin (2020) [47] | Retro-spective cohort | craniotomy tumor resection | Meningioma, not returning (n=12)/ returning (n=19) to work; Glioma, not returning (n=20)/ returning to work (n=9) | 60 | 12-item | Self-reported | Not described, no deaths | 18% |
| Schiavolin (2022) [48] | Prospective cohort | craniotomy | Glioma  (n=64)/ meningioma (n=85) | 149 | 12-item | Self-reported | Excluded from analysis | Unclear |
| Short (2019) [49] | RCT | surgery with general anaesthesia | Light general anaesthesia (BIS 50) (n=3316)/ deep general anaesthesia (BIS 30) (n=3328) | 6644 | 12-item | Interview and self-reported | Multiple scenario’s | 1% |
| Shulman (2019) [50] | Prospective cohort | ambulatory, intermediate, major noncardiac, cardiac, or nonelective surgery | Total population | 574 | 12-item | Not specified | Not described / available data analysis | 0.3%-10% |
| Smith (2022) [51] | Prospective cohort | major surgery | Completed 6-MWT (n=229)/ Non-completion 6-MWT (n=64) | 293 | 12-item | Not specified | Not described, no deaths | 0%-5% |
| Spann (2019) [52] | Prospective cohort | Bariatric surgery | Total population | 110 | 12-item | Not specified | Not described, no deaths | 0%-51% |
| Suzuka (2024) [53] | RCT | Elective gastrectomy (robot-assisted vs laparoscopic) (cancer surgery) | external oblique intercostal block (n=15)/ wound infiltration (n=17) | 32 | Not specified | Not specified | Not described, no deaths | 0%-0% |
| Tanaka (2024) [54] | RCT | robot-assisted laparoscopic radical prostatectomy (cancer surgery) | Nociception level-directed analgesic management (n=25)/ conventional analgesia management (n=25) | 50 | 12-item | Self-reported | Not described, no deaths | 2% |
| Vail (2024)* [55,56] | RCT | surgical repair of a femoral neck, intertrochanteric, or subtrochanteric hip fracture. | Spinal anaesthesia (n=795)/ general anaesthesia (n=804) | 1600 | 12-item | Interview of patient or proxy | Not described, death occurred | 0.1%-71% |
| Vernooij (2024) [57] | Prospective cohort | elective major noncardiac surgery | Total population | 888 | 12-item | Interview | Scored 100% | 8%-9% |
| White (2018) [58] | Prospective cohort | Surgical intervention | Patients with complete (n=173) /incomplete¶ (n=228) follow up | 401 | 12-item | Interview | Not described, no deaths | 0%-56% |
| Yabuno (2025)* [59,60] | Prospective cohort | noncardiac surgery under general anaesthesia | Total population | 2596 | 12-item | Self-reported | Death excluded | 31% |

* Data available from merged studies[7,24,55,59]**; ****12-item calculated; † adjusted to 32 questions; ‡not specified but referred to Shulman 2015; § described as minimum-maximum percentage missing or overall percentage missing, depending on data available; ¶ Not included in primary analysis due to missing postoperative WHODAS 2.0 scores; †† exclusion of 20/75 patients due to incomplete data; ‡‡ 142 patients (70 prehabilitation; 72 usual care) did not have surgery, but were included in WHODAS 2.0 analyses, 62% of all patients had cancer surgery; §§ 8 patients (2 no prehabilitation; 6 prehabilitation) did not have surgery, but were included in WHODAS 2.0 analyses; ¶¶ laparoscopic colorectal surgery for cancer for 20/24 patients;

Table S2: Population characteristics of included studies

| **Surgery** | **Author (Year)** | | **Total sample**  **size** | **Average patient age (years)** | **Male gender**  N (%) | **ASA** N (%) | | | | **Preoperative disability (>25%)**  N (%) |
| --- | --- | --- | --- | --- | --- | --- | --- | --- | --- | --- |
|  |  |  |  |  |  | I | II | III | IV |  |
| Mixed | Admiraal (2023) | 176 | | 54.1 (15.1);  53.8 (15.1) | 50 (28.4%) | 21 (11.9%) | 82 (46.6%) | 72 (40.9%) | 1 (0.6%) |  |
|  | Daza (2024) θ | 3000 | | 66 (13) | 1554 (51.8%) | 46 (1.5%) | 1082 (36.1%) | 1717 (57.2%) | 138 (4.6%) |  |
|  | Ferraz (2018) | 182 | | 63 (47-70);  56 (54-64) | 62 (34.1%) | 21 (11.5%) | 128 (70.3%) | 32 (17.6%) | 1 (0.5%) | 22 (12%), N=182 |
|  | Kant (2025) | 3247 | | 59 (44-69); 59 (45-69) | 1509 (46%) | 552 (17.0%) | 1873 (57.7%) | 822 (25.3%) | |  |
|  | McIsaac (2020) | 702 | | 73 (6) | 353 (50.3%) |  |  |  |  |  |
|  | Posthuma (2022) | 757 | | 60.1 (13.1);  58.8 (14.6) | 441 (58.3%) | 69 (9.1%) | 463 (61.2%) | 210 (27.7%) | 5 (0.7%) | 73 (10%)*, N=742 |
|  | Sato (2025) | 46 | | 72 (69-75) |  | 5 (10.9%) | 35 (76.1%) | 6 (13.0%) |  |  |
|  | Short (2019) | 6644 | | 72 (7) | 4221 (63.5%) | 0 (0%) | 0 (0%) | 6302 (94.9%) | 341 (5.1%) |  |
|  | Shulman (2019) | 574 | | 65 (57-72) | 351 (61.1%) | 22 (3.8%) | 321 (55.9%) | 222 (38.7%) | 8 (1.4%) | 103 (18%), N=572 |
|  | Smith (2022) | 293 | | 58 (13.8) | 123 (42%) | 6 (2%) | 145 (49.5%) | 130 (44.4%) | 6 (2%) | 99 (34%), N=293 |
|  | Vernooij (2024) | 902 | | 69.0 (53–91) † | 532 (59%) |  |  |  |  | 90 (10%)*, N=888 |
|  | White (2018) | 401 | | 35 (20-49);  31 | 187 (46.6%) | 267 (66.6%) | 111 (27.7%) | 7 (1.7%) | 0 (0%) | 68 (17%), N=401 |
|  | Yabuno (2025) | 2596 | | 69.0 (64.0–74.0) | 1399 (53,9%) | 292 (11.2%) | 2057 (79.2%) | 240 (9.2%) | 7 (0.3%) |  |
| Bariatric | Spann (2019) | 110 | | 43.9 (10.5) | 27 (24.5%) |  |  |  |  |  |
| Cardio-vascular | Haakseth (2023) | 25 | | 73.2 (7.7) | 18 (72%) |  |  |  |  |  |
|  | Lee (2025) †† | 143 | | 64 (58-69); 65 (60-71) | 101 (70.6%) |  | 10 (7.0%) | 118 (82.5%) | 6 (4.2%) | 16 (12%), N=133 |
|  | Ma  (2025) | 351 | | 66.8 (5.2) | 232 (66.1%) |  |  |  |  | 70 (20%), N=351 |
|  | Miles (2022) | 480 | | 62.9 (12.2);  64.3 (10.8) | 325 (67.7%) |  |  |  |  |  |
|  | Milne (2022) | 146 | | 66.0 (58.0-72.5); 71.0 (59.5–77.0) | 107 (73.3%) | - | - | 101 (69,2%) | 45 (30,8%) | 42 (28%), N=146 |
|  | Parke (2021) | 715 | | 68 (59–74); 70 (59–74) | 526 (73.6%) |  |  |  |  | 131 (25%), N=535 |
| Neuro-surgery | Schiavolin (2018) | 101 | | 50.2 (14.09) | 47 (46.5%) |  |  |  |  |  |
|  | Schiavolin (2020) | 60 | | 48.1 (8.0);  46.0 (11.8) | 27 (45%) |  |  |  |  |  |
|  | Schiavolin (2022) | 149 | | 50 (17.0); 55 (11.3) | 57 (38.3%) |  |  |  |  |  |
| Cancer | Amirpour (2025) ‡‡ | 24 | | 77 (6.5) | 11 (46%) | 4 (17%) | 11 (46%) | 8 (33%) |  |  |
|  | Bobcikova (2023) | 44 | | 65.7 (9.3) | 25 (56.8%) |  |  |  |  |  |
|  | Ida (2019) | 24 | | 69.6 (7.5) | 17 (70.8%) |  |  |  |  |  |
|  | Catto (2022) | 317 | | 69.3 (8.0);  68.7 (8.4) | 250 (74%) |  |  |  |  |  |
|  | Collange (2024) ‡ | 155 | | 66 (14);  64 (13) | 78 (50.3%) | 26 (16.8%) | 79 (51%) | 46 (29.7%) | 0 (0%) |  |
|  | Kinugasa (2023) | 260 | | 73 (69-77) | 161 (61.9%) | 9 (3.5%) | 178 (68.5%) | 42 (16.2%) | 1 (0.4%) | 33 (14%)**, N=230 |
|  | Liu (2020) | 73 | | 56.2 (10.3);  56.2 (8.7) | 23 (31.5%) |  |  |  |  |  |
|  | McIsaac (2022) | 204 | | 74 (6); 74(7) | 79 (38.7%) | 150 (73.5%) | | | 32 (15.7%) |  |
|  | McIsaac (2025) ¶ | 847 | | 71.7 (7.0); 71.7 (7.2) | 395 (46.6%) |  |  |  |  |  |
|  | Miles (2025) | 420 | | 63 (54–70);  64 (54–70) | 272 (65.4%) |  |  |  |  |  |
|  | Suzuka (2024) | 32 | | 68.2 (10.5);  70.6 (8.3) | 25 (78.1%) | 2 (6.2%) | 30 (93.8%) | 0 (0%) | 0 (0%) |  |
|  | Tanaka (2024) | 50 | | 68 (6.6);  68 (5.8) | 50 (100%) | 6 (12%) | 43 (86%) | 1 (2%) | 0 (0%) |  |
| Ortho-paedic | Edgley (2019) | 303 | | 44 (30-58);  40 (28-55) | 212 (70%) | 250 (82,5%) | | 53 (17,5%) | | 272 (90%), N=303 |
|  | Galli (2018) | 80 | | 70.1 (0.7) | 25 (31.2%) |  |  |  |  |  |
|  | Schiavolin (2015) | 75 | | 54.4 (12.2) | 32 (42.7%) |  |  |  |  |  |
|  | Vail (2024) § | 1600 | | 77.7 (10.7);  78.4 (10.6) | 528 (33%) | 499 (31.2%) | 949 (59.3%) | 87 (5.4%) |  |  |

Average patient age is described as mean (standard deviation) or median (1^st^ interquartile – 3^rd^ interquartile) unless otherwise stated; *disability defined >35%; ** disability defined >16%; † interquartile range; ‡ colorectal resection due to non-cancer indication for 75/155 patients); § data acquired from merged studies [24,25,55,56]; ¶ 142 patients (70 prehabilitation; 72 usual care) did not have surgery, but were included in WHODAS 2.0 analyses, 62% of all patients had cancer surgery; †† 8 patients (2 no prehabilitation; 6 prehabilitation) did not have surgery, but were included in WHODAS 2.0 analyses; ‡‡ laparoscopic colorectal surgery for cancer for 20/24 patients;

Table S3: Surgical procedure types in studies with mixed surgical populations

| **Study** | **Inclusion criteria** | **Surgical types** n (%) |
| --- | --- | --- |
| Admiraal (2023) | Patients with an increased risk of chronic postsurgical pain, planned for elective surgery | Amputation 3 (3.6); 1 (1.1)  Hip arthroplasty 21 (25.0); 20 (21.7)  Hysterectomy 15 (17.9); 18 (19.6)  Knee arthroplasty 10 (11.9); 13 (14.1)  Thoracotomy 1 (1.2); –  Other 34 (40.1); 40 (43.4) |
| Daza (2024) | Adults who had an increased risk of complications while undergoing major abdominal surgery that included a skin incision, an expected operative duration of at least 2 hours, and an expected hospital stay of at least 3 days. | Oesophageal or gastric 286 (19.2); 257 (17.2)  Hepatobiliary 133 (8.9); 139 (9.3)  Colorectal 646 (43.4); 651 (43.6)  Urologic or renal 220 (14.8); 223 (14.9)  Gynaecologic 135 (9.1); 139 (9.3)  Other 70 (4.7); 84 (5.6) |
| Ferraz (2018) | Undergoing non-cardiac, non-obstetric, and non-neurological surgery; and those being admitted to the PACU | Gynaecologic 32 (18)  Head and neck 13 (7)  Gastrointestinal 82 (45)  Urologic 10 (5)  Breast 32 (18)  Plastic and reconstructive 13 (7) |
| Kant (2025) | Elective noncardiac surgery under general or central-neuraxial anesthesia with an expected hospital stay of at least 1 night after surgery | General 526 (32.5); 462 (28.4)  Gynaecologic 247 (15.3); 291 (17.9)  Orthopaedic 227 (14.0); 242 (14.9)  ENT or head and neck 228 (14.1); 216 (13.3)  Neurosurgery 191 (11.8); 198 (12.2)  Urologic 146 (9.0); 160 (9.8)  Plastic 53 (3.3); 59 (3.6) |
| Kinugasa (2023)** | Aged 65 years and older, undergoing elective major abdominal surgery (general, urologic, and gynaecologic surgery) with a cancer diagnosis associated with a reduced likelihood of disability-free survival | General 167 (72.6)  Urologic 57 (24.7)  Gynaecologic 6 (2.6) |
| McIsaac (2020) | Elective noncardiac surgery with expected length of stay of ≥2 days | Orthopaedic 143 (56.8); 209 (48.1)  Abdominal 36 (14.3); 102 (23.5)  Thoracic 25 (9.9); 43 (9.9)  Neurosurgery 15 (6.0); 19 (4.4)  Vascular 33 (13.1); 62 (14.3) |
| McIsaac (2022)** | Aged 60 years or older; scheduled to undergo elective surgery for intra-abdominal and intrathoracic cancer (colorectal, thoracic, hepatobiliary or urological); able to communicate in French or English; willing to participate in home-based exercise and identified with frailty based on the Clinical Frailty Scale (CFS; score of > 4/9) | Colorectal 22 (25.0); 36 (38.3)  Hepatobiliary 25 (28.4); 21 (22.3)  Thoracic 37 (42.1); 33 (35.1)  Urologic 4 (4.6); 4 (4.3) |
| McIsaac (2025)** | Aged 60 years or older, living with frailty, scheduled for elective, inpatient head and neck, thoracic, abdominal, pelvic, or vascular surgery | Colorectal 92 (21.8); 89 (21.0)  Head and neck 11 (2.6); 18 (4.3)  Hepatobiliary 57 (13.5); 49 (11.6)  Thoracic 68 (16.1); 71 (16.8)  Vascular 55 (13.0); 53 (12.5)  Urologic or gynaecologic 94 (22.2); 103 (24.3)  Other general surgery 46 (10.9); 41 (9.7) |
| Posthuma (2023) | Non-cardiac intermediate or high-risk  surgery | Severity of surgery, n ()  Minor 0 (0); 1 (0.4)  Intermediate 236 (45.6); 108 (47.0)  Major 281 (54.4); 121 (52.6) |
| Sato (2025) | Scheduled for urological abdominal, lower gastrointestinal, thoracic surgery, or oral and maxillofacial surgery requiring vascular reconstruction | Urological abdominal 24 (52.2)  Thoracic 17 (37)  Gastrointestinal lower abdominal 3 (6.5)  Oral surgery involving vascular reconstruction 2 (4.3) |
| Short (2019) | 1. Age 60 years and over; 2. ASA physical status 3 or 4; 3. General anaesthesia (±major regional anaesthesia); 4. Anticipated duration of anaesthesia >2 hours; 5. Anticipated duration of postoperative hospital stay ≥2 nights; | Cardiac 53 (2); 60 (2)  Head and neck 77 (2); 86 (3)  Intra-abdominal 1528 (46); 1525 (46)  Orthopaedic 361 (11); 344 (10)  Spinal 267 (8); 249 (7)  Thoracic 234 (7); 234 (7)  Vascular 634 (19); 649 (20)  Other 162 (5); 181 (5) |
| Shulman (2019) | Aged 40yrs or older, scheduled for elective non-cardiac surgery with a minimum of an overnight hospital stay, and had at least one cardiac risk factor. | Orthopaedic 88 (16)  Urologic or gynaecologic 157 (29)  Intraperitoneal or retroperitoneal 179 (33)  Head and neck surgery 57 (11)  Intrathoracic 22 (4)  Vascular 17 (3)  Other surgery 22 (4) |
| Smith (2022) | Undergoing any type of booked elective surgery, with a BMI of 35 kg/m2 | Orthopaedic 90 (31)  Urology 55 (19)  Gynaecology 46 (16)  General 43 (15)  ENT and maxillofacial 17 (6)  Neurosurgery 16 (5)  Vascular 13 (4)  Colorectal 6 (2)  Plastic 5 (2)  Other 2 (1) |
| Vernooij (2004) | Elective major noncardiac surgery under general or spinal anaesthesia with an expected postoperative hospital stay exceeding 24 h | ENT and dental 131 (14.8)  General 180 (20.3)  Neurosurgery 60 (6.8)  Orthopaedic 89 (10.0)  Thoracic 45 (5.1)  Urological / gynaecological 179 (20.2)  Vascular 204 (23.0) |
| White (2018)* | Patients undergoing surgery | General 61 (35)  Maxillofacial 48 (28)  Orthopaedic 28 (16)  Plastics 10 (6)  Women’s Health 26 (15) |
| Yabuno (2025) | Patients with elective surgery under general anaesthesia and WHODAS value at 5 years | Orthopaedic surgery 346 (13.3)  Non-orthopaedic 2250 (86.3) |

* n(%) of patients with WHODAS 2.0 scores available (n=173); ** categorised as cancer surgery (>50% cancer surgery)

Table S4: Preoperative and postoperative average WHODAS 2.0 metric scores per study

|  |  |  |  | **Measurement after surgery** | | | | |
| --- | --- | --- | --- | --- | --- | --- | --- | --- |
| **Surgical specialty** | **Study** | **cohorts** | **Before surgery *** | **1**  **month** | **2 months** | **3**  **months** | **6**  **months** | **12 months** |
| Cardio-Vascular | Haakseth (2023) | paired baseline to 1-month | 17.0 (13.4) | 29.6 (22.4) |  |  |  |  |
|  | Lee (2025) | All patients | 14.1 (13.4) |  |  | 10.4 (24.1) |  |  |
|  | Ma (2025) | All patients | 5 (2–9) |  |  | 3 (1–7) |  |  |
|  | Miles (2022) | Iron deficient group | 8 (2–17) |  |  | 8.3 |  |  |
|  |  | Iron replete group | 4 (0–15) |  |  | 7.0 |  |  |
|  | Milne (2022) | All patients | 12.5 (6.3–26.6) | 19.8 (6.3–37.0) |  | 4.2 (0.0–16.7) |  |  |
|  | Parke (2021) | Usual care | 16.0 (‡) |  |  | 8.9 (‡) | 7.0 (‡) |  |
|  |  | Fluid administration protocol | 15.7 (‡) |  |  | 8.5 (‡) | 7.2 (‡) |  |
| Neuro-surgery | Schiavolin (2018) | all patients | 24.3 (18.4) |  |  | 25.4 (20.5) |  |  |
|  | Schiavolin (2020) | Meningioma - Return to work | 19.0 (16.9) |  |  | 12.6 (12.5) |  |  |
|  |  | Meningioma - Not return to work | 20.6 (17.7) |  |  | 29.0 (21.9) |  |  |
|  |  | Glioma - not return to work | 7.6 (6.0) |  |  | 7.4 (8.4) |  |  |
|  |  | Glioma - Return to work | 18.2 (17.4) |  |  | 27.3 (18.2) |  |  |
|  | Schiavolin (2022) | Glioma | 18.1 (‡) |  |  | 23.6 (‡) |  |  |
|  |  | Meningioma | 19.0 (‡) |  |  | 18.7 (‡) |  |  |
| Cancer | Amirpour (2025) | All patients | 9.4 (9.6) | 18 (17.1) |  |  | 14.4 (13.1) |  |
|  | Bobcikova (2023) | All patients | 6.1 (6.5) |  |  | 10.2 (8.7) |  |  |
|  | Catto (2022) | Robotic cystectomy | 9.3 (12.4) | 20.9 (18.2) |  | 10.2 (11.5) | 10.8 (13.3) | 10.8 (15.8) |
|  |  | Open cystectomy | 9.2 (11.4) | 26.4 (18.7) |  | 14.9 (16.3) | 11.1 (13.1) | 10.1 (14.4) |
|  | Collange (2024) | Opioid-free anaesthesia | 10 (4–17) | 8 (0–23) |  | 2.1 (0–15) |  |  |
|  |  | Opioid-sparing anaesthesia | 6 (4–21) | 8 (2–19) |  | 6 (0–10) |  |  |
|  | Ida (2019) | All patients | 33.3 (4.1**] |  |  | 37.5 (4.8**] |  |  |
|  | Kinugasa (2023) | All patients | 4.2 (0–17.3] |  |  | 8.5 (0–30.2] |  |  |
|  | Liu (2020) | Prehabilitation program) | 4 (2–6) | 6 (2–12) |  |  |  |  |
|  |  | Standard care | 4 (2–7) | 10 (4–15) |  |  |  |  |
|  | McIsaac (2022) | Usual care | 18.6 (13.7) | 26.8 (19.2) |  |  |  |  |
|  |  | Prehabilitation | 19.0 (13.7) | 24.8 (16.8) |  |  |  |  |
|  | McIsaac (2025) | Usual care | 14.0 (11.9) | 24.7 (23.8) |  |  |  |  |
|  |  | Prehabilitation | 13.5 (11.9) | 23.5 (21.8) |  |  |  |  |
|  | Miles (2025) | Iron deficient | 4 (2–13) |  |  | 4 (‡) |  |  |
|  |  | Iron replete | 4 (2–10) |  |  | 4 (‡) |  |  |
|  | Suzuka (2024) | External oblique intercostal block | 4.7 (4.4) |  |  | 11.3 (17.5) |  |  |
|  |  | Wound infiltration | 10.7 (16.2) |  |  | 16.8 (19.3) |  |  |
|  | Tanaka (2024) | Nociception level-directed analgesic management | 4.3 (7.1) |  |  | 9.3 (11.8) |  |  |
|  |  | Conventional analgesia management | 5.0 (5.2) |  |  | 9.8 (10.0) |  |  |
| Ortho-paedic | Edgley (2019) | No severe acute pain at PACU | 56 (44–63) |  |  | 19 (4–35) |  |  |
|  | Edgley (2019) | Severe acute pain at PACU | 54 (40–63) |  |  | 29 (8–50) |  |  |
|  | Galli (2018) | All patients | 37.3 (9.1) | 15.7 (11.7) |  |  |  |  |
|  | Vail (2024) | Spinal anesthesia | 9.1 (2.1–22.9) |  | 22.7 (8.3–43.2) |  | 10.4 (2.3–27.1) | 10.4 (2.1–29.2) |
|  | Vail (2024) | General anesthesia | 8.3 (2.1–25.0) |  | 18.2 (6.3–31.8) |  | 10.4 (2.1–27.3) | 8.3 (0–22.9) |
|  | Schiavolin (2015) | All patients | 34.1 (17.9) |  |  | 24.5 (17.1) |  |  |
| Bariatric | Spann (2019) | All patients | 13.9 (12.7) | 8.4 (‡) | 5.6 (‡) | 5 (‡) |  |  |
| Mixed | Admiraal (2023) | Standard care | 61.4 (21.4) † |  |  | 53.1 (20.5) † | 49.6 (21.2) † |  |
|  |  | Transitional pain service | 61.4 (20.5) † |  |  | 49.8 (18.7) † | 47.0 (21.3) † |  |
|  | Daza (2024) | Liberal | 17.6 (6.9) |  |  |  |  | 17.8 (8.4) |
|  |  | Restrictive | 17.9 (6.7) |  |  |  |  | 17.8 (8.4) |
|  | Ferraz (2018) | With Postoperative Qualitative Recovery | 5.2 (4.2–13.0) |  |  | 2.1 (0–4.2) |  |  |
|  |  | Without Postoperative Qualitative Recovery | 2.1 (0–8.3) |  |  | 2.1 (0–6.3) |  |  |
|  | Kant (2025) | Proactive blood pressure management | 12.5 (4.2-29.2) | 22.9 (10.4-39.6) |  |  | 17.7 (20.1) |  |
|  |  | Usual management | 14.6 (4.2-29.2) | 25 (10.4-41.7) |  |  | 17.7 (20.1) |  |
|  | McIsaac (2020) | No frailty | 13.3 (22.9) |  |  |  |  | 14.1 (‡) |
|  |  | Frailty | 26.9 (24.6) |  |  |  |  | 19.6 (‡) |
|  | Posthuma (2022) | All patients | 8.3 (16.7**) | 22.9 (29.2**) |  | 12.5 (18.8**) |  |  |
|  | Sato (2025) | All patients | 2.1 (0-6.8) |  |  | 6.3 (2.1-16.7) |  |  |
|  | Short (2019) | Light general anaesthesia (BIS 50) | 18 (14–25) | 18 (14–25) |  |  |  | 16 (13–23) |
|  |  | Deep general anaesthesia (BIS 35) | 18 (14–25) | 18 (13–25) |  |  |  | 16 (12–23) |
|  | Shulman (2019) | All patients | 8 (2–19) |  |  |  |  | 4 (0–13) |
|  | Smith (2022) | non-completion 6-Minute walking test | 31.0 (15.1) |  |  |  | 13.0 (17.1) |  |
|  | Smith (2022) | completed 6-Minute walking test | 15.0 (15.5) |  |  |  | 6.0 (10.6) |  |
|  | Vernooij (2024) | All patients | 6.3 (2.1–20.8) |  |  |  | 6.3 (0–20.8) |  |
|  | White (2018) | Patients followed up with WHODAS 2.0 | 8.36 (0.09) |  |  | 1.00 (0.01) |  |  |
|  | Yabuno (2025) | All patients | 6.25 (2.0–16.6) |  |  |  |  | 8.3 (2.0-22.9) ¶ |

*Data is displayed as mean (standard deviation) or median (1^st^ interquartile – 3^rd^ interquartile) unless otherwise stated. **Interquartile range; † Standard deviation is estimated following previous recommendations [61]; ‡information on the spread of data not available; ¶ measurement at 60 months after surgery;
